# Fear Related to COVID-19, Mental Health Issues, and Predictors of Insomnia among Female Nursing College Students during the Pandemic

**DOI:** 10.3390/healthcare11020174

**Published:** 2023-01-06

**Authors:** Zainab Fatehi Albikawi

**Affiliations:** Community and Psychiatric/Mental Health Nursing Department, Nursing College, King Khalid University, Khamis Mushait 39746, Saudi Arabia; zalbikawi@kku.edu.sa; Tel.: +966-5-6112-8667

**Keywords:** fear related to COVID-19, depression, insomnia, anxiety, chronic illnesses, family support

## Abstract

Fear of infection has been sparked by the advent of the novel coronavirus disease (COVID-19). Insomnia in college students, especially its correlations and predictions with mental diseases, remains a research concern. Aim: To estimate the prevalence of fear related to COVID-19, depression, anxiety, and insomnia among female nursing college students throughout the pandemic and to determine the predictors of insomnia. Methods: A web-based cross-sectional descriptive study used 145 female nursing college students. Results: Students reported fear related to COVID-19, depression, and anxiety at rates of 79.3%, 30.2%, and 35.2%, respectively. Insomnia disturbed 24.7% of students. Anxiety predicted worsening insomnia in the student (AOR = 1.08, 95% CI: 0.92–0.97, *p* < 0.001). Fear related to COVID-19 was also a predictor (AOR = 0.96, 95% CI: 1.07–1.21, *p* < 0.05). Additionally, when depression severity declined, the chance of insomnia improved (AOR = 0.87, 95% CI: 0.85–0.91, *p* < 0.001). Insomnia was more common in chronically unwell students (AOR = 1.50, 95% CI = 1.01–2.24, *p* < 0.05). Conclusion: During the COVID-19 pandemic, university students’ mental health should be monitored, and all essential safeguards should be taken, including resource allocation, awareness raising efforts, and the building of a mental health counseling facility.

## 1. Introduction

The pandemic caused by SARS-CoV-2 (COVID-19) is the most serious threat to public health that the world has been confronted with in recent memory. The mental health issues studied during the pandemic have been the subject of a number of studies, which have focused on different populations such as healthcare professionals [1], college students [2], and adults [3]. In this perspective, college students are an especially susceptible group to the development of mental health issues such as depression, anxiety [4], and insomnia [5], because they are typically experiencing a period of transition in their academic, career, and personal lives [6]. Mental-health-related concerns are made more complicated for nursing students who aspire to become healthcare professionals in the future [7].

### 1.1. Mental Health Issues, Fear Related to COVID-19, and Insomnia

Anxiety and depression were among the mental-health-related symptoms explored in prior studies conducted on nursing students during the pandemic. The prevalence of these symptoms was reported to be between 30% and 50% [8,9,10]. Several studies on nursing students’ rates of depression and anxiety disorders have been carried out during and before the pandemic [11,12]. The prevalence of depression and anxiety disorders among nursing students had been high, reaching 48.5% and 37.3%, respectively, during the COVID-19 pandemic, according to a number of recent studies [11,12]. In studies which were quite comparable to one another, researchers in China, Japan, and Saudi Arabia found that nursing students exhibited a significant prevalence of depression (56.4%, 31.1%, and 43.3%, respectively) and anxiety (55.0%, 30.5%, and 37.2%, respectively) [8,9,13]. Patterns and clinical characteristics of depression and anxiety may vary depending on a sample’s characteristics, including age, culture, socioeconomic factors, the presence or absence of medical conditions, and specific stressors [14,15,16]. 

Fear related to COVID-19 is mental construct known as coronaphobia, defined as a severe fear of contracting COVID-19, which results in physical symptoms such as palpitations, trembling, shortness of breath, dizziness, change in appetite, and insomnia, as well as catastrophic thoughts that elicit negative emotions such as sadness, guilt, and rage. All of this leads to avoidance habits, which decrease the quality of life for the individual [17]. Fear of infection, difficulties with clinical practical training, damaged interpersonal relationships, and fear over academics and career are some of the negative outcomes that nursing students have experienced during the COVID-19 pandemic [18]. It was discovered that this kind of fear had a negative impact not only on the nursing students’ college life, but also on their educational performance and their ability to pass the nurse licensure examination [19]. In addition, there was an 86.0% prevalence of fear related to COVID-19 among college students during the pandemic [20]. 

Insomnia has emerged as one of the most significant mental problems during the pandemic [21]. It is the most common form of sleep problem that affects the general population, and it is a major cause for concern when it comes to a person’s health. It is a long-term condition that manifests itself as trouble falling asleep, an increased number of awakenings throughout the night, difficulty falling back to sleep, and waking up earlier than expected [22]. The prevalence of insomnia among college students has been the subject of a number of studies, carried out prior to the pandemic in a variety of nations; the findings have revealed a wide range of incidence rates. In Lebanon, Italy, and Poland, there is a low prevalence rate that affects less than 20% of the student population [23,24,25].

In certain countries before the pandemic, such as the United States, Jordan, Saudi Arabia, Norway, and China, the prevalence of insomnia was only moderate, ranging from 20% to 40% [26,27,28]. In Canada, Ethiopia, and Hong Kong, a startlingly high prevalence of insomnia has been observed, with between 50% and 70% of the students being affected [29,30,31]. Notably, female students are more likely to be affected by insomnia than male students [30,32]. During the pandemic, the proportion of college students in Argentina who suffered from insomnia was 27%.

### 1.2. Factors Associated with Insomnia

Multiple causes of insomnia have been found. The majority of young adults acquire insomnia throughout their college years [31]. Insomnia is the difficulty of falling asleep which is caused by anxiety and is brought on by agitation. Consequences of the vicious cycle include chronic insomnia, continuous fatigue, and even depressed symptoms. Age, stress, stimulants, physical activity, and habits are just a few of the numerous factors that may contribute to insomnia and other sleep disorders [33]. In addition, there is a higher incidence of insomnia among students who struggle with mental health conditions such as anxiety and depression [31,34,35]. A study conducted in China during the pandemic revealed that young individuals have significant rates of anxiety and depression, which affects their ability to sleep [36].

### 1.3. Female Nursing College Students and the Study Variables

In the field of nursing, women typically outnumber men; approximately 75% of Saudi Arabia’s nursing workforce is female [37]. Nursing students may be ignorant of or misinformed about diseases and infection prevention techniques that are pertinent to clinical practice [38], which may result in greater infection transmission and may exacerbate the psychological suffering of students [39]. A study was carried out in Saudi Arabia in April 2020 to evaluate the pandemic’s effects on university students’ mental health [40]. Among Saudi university students, 40.8%, 48.8%, and 86.7% indicated anxiety, depression, and moderate to high stress symptoms, respectively. These symptoms were significantly more common in females and younger individuals [40]. A cross-sectional study of 790 participants during the COVID-19 quarantine in Saudi Arabia indicated that 55.5% had poor sleep quality and 54.4% had insomnia. Additionally, marriage and feminine gender were risk factors [41]. Both before [42,43] and during COVID-19, females display more severe sleep issues and depressive symptoms [44].

In the context of the Middle East, particularly in Saudi Arabia, researchers have explored how common mental health disturbances are among healthcare personnel [45,46,47,48]. Not much research has assessed students’ mental health during the pandemic [49,50], especially nursing university students [8]. The vast majority of studies just describe the symptoms of mental illness (such as anxiety, depression, insomnia, and stress). Additionally, during the COVID-19 pandemic, there has reportedly been a greater psychological impact on female college students [51]. However, the literature on mental effects, fear related to COVID-19, and insomnia in female nursing college students is scarce. Therefore, this study was carried out to fulfill two distinct aims: to estimate the prevalence of fear related to COVID-19, depression, anxiety, and insomnia among female nursing college students throughout the pandemic; and to determine the predictors of insomnia.

## 2. Materials and Methods

### 2.1. Design

Descriptive and web-based cross-sectional research methods were used in this study. The decision to carry out a cross-sectional study was driven by the fact that this type of research yields results on a sizable number of participants in a short period of time. Saudi Arabian female nursing college students participated in the study.

### 2.2. Sample and Study Population

The sample size estimate performed using G Power 3.1.9.2 indicated that a minimum of 150 participants were needed to conduct a study with a significance level of 0.05 and a statistical power of 0.95. Using a convenience sample technique, 150 female nursing college students in Saudi Arabia were chosen. To take part in the study, participants had to be enrolled in a nursing program and be competent to give informed consent. Participants who had a diagnosis of any psychiatric or mental health illnesses or who were enrolled in another medical field were excluded from the study (assessed through socio-demographic questionnaires).

### 2.3. Data Collection

The information was gathered from female nursing students between March 10 and April 28, 2021. A notice about the study and a link to the tools were published together. Along with the link to the study materials, a cover letter outlining the goals of the study and assuring participants’ confidentiality and anonymity was also posted. The link to fill out the Google Form with the questionnaire was clicked by students who agreed to participate in the study. Self-reporting questionnaires, including the sociodemographic questionnaire, the WHO-Five Well-being Index (WHO-5), the Generalized Anxiety Disorder (GAD-7), the Fear of COVID-19 scale (FCV–19S), and the Bergen Insomnia Scale, were utilized to collect data for the current study. Nursing students studying for a bachelor’s degree were able to read and write English; therefore, the scales were given to the students along with their original copies.

#### 2.3.1. Sociodemographic Questionnaire

This questionnaire was developed by the researcher, and the closed-ended questions in this part were generated from prior empirical findings of factors with a substantial association with insomnia, anxiety, fear related to COVID-19, and depression. The author made the decision to divide the participants into two groups according to their ages. The first group, which ranged from 18 to 22 years old, is primarily the age of undergraduate nursing students who are in their first to fourth year of college. The second group, which was equal or older than 23 years old, consisted of students who had already begun their clinical training in internship. In addition, previous research has revealed that the psychological consequences of the pandemic are more severe for young adults when compared with older adults [3,52,53]. Marital status, monthly family income, and the existence of chronic diseases such as diabetes, hypertension, and heart diseases were included in the questionnaire.

Additionally, family support was assessed by asking the students, “Do you believe your family is supporting you?”. The researcher did not use a valid tool to measure family support because it was not included in the main study objective. However, the reason to include such a question was to emphasize the importance of family support for enhancing the mental health of nursing students and to give further direction for assessing the impact of family support on nursing students’ mental health.

#### 2.3.2. WHO-Five Well-Being Index (WHO-5)

The WHO-5 is a five-item self-report questionnaire that rates well-being on a 6-point Likert scale (0 being “not present” to 5 being “constantly present” over the course of the previous two weeks. Higher WHO-5 scores, which range from 0 to 25, represent better well-being. A cut-off score of 12 or less suggests poor health and the need for additional depression testing. In various countries across Africa, Asia, Australia, Europe, North America, and South America, the WHO-5 has shown strong reliability and validity and the capacity to identify persons with depression [54].

#### 2.3.3. The Bergen Insomnia Scale (BIS)

Diagnostic criteria from the Diagnostic and Statistical Manual of Mental Disorders formed the basis of this instrument. It comprises six items, with the first three discussing morning insomnia and the onset of sleep. The final three elements are dissatisfaction with present sleep patterns, insufficient rest, and impairment during the day. On a scale from 0 to 7 on an 8-point scale, the number of sleepless nights each week is recorded [55].

#### 2.3.4. The Generalized Anxiety Disorder (GAD-7)

Using the Generalized Anxiety Disorder 7-Item Scale (GAD-7), anxiety symptoms were assessed. Spitzer et al. (2014) created the GAD-7, a seven-item anxiety scale with a score ranging from 0 to 3 for each item. The GAD-7 scale has a range of 0 to 21, with a total score between 0 and 4 indicating no symptoms of anxiety, 5 to 9 indicating mild anxiety, 10 to 14 indicating moderate anxiety, and 15 or more indicating severe anxiety. The factorial validity and reliability of the GAD-7 scale were good (Cronbach’s alpha = 0.901) [56].

#### 2.3.5. Fear of COVID-19 Scale (FCV-19S)

The Fear of COVID-19 scale (FCV-19S) was used to measure fear related to COVID-19. A five-point Likert-type scale with seven items, the FCV-19S, featured responses such as “1 = strongly disagree”, “2 = disagree”, “3 = neither agree nor disagree”, “4 = agree”, and “5 = strongly agree”. Higher scores, ranging from 7 to 35, indicate greater anxiety of COVID-19. A higher score denotes a stronger fear related to COVID-19 in the current study, which used the seven-item, unidimensional construct with four-point Likert-type answers [57].

### 2.4. Ethical Considerations

The study was approved by the Institutional Review Board (IRB). The study’s participants, female nursing students, were made aware that their participation was completely optional and that they might discontinue it at any time by deciding not to respond to any questions. To preserve their privacy, the results would only be shared in aggregate.

### 2.5. Statistical Analysis

In three distinct processes, version 20 of the Statistical Program for the Social Sciences (SPSS) was utilized to examine the data. First, descriptive statistics were employed to illustrate data regarding the individuals’ mental health issues (depression and anxiety), fear related to COVID-19, and insomnia. These statistics comprised frequency and percentage analyses in addition to measures of central tendency (such as mean and standard deviation). Second, bivariate analyses were undertaken using *t*-tests (*t*) carried out in order to investigate the possibility of substantial mean differences between demographics and insomnia. Third, Pearson correlation (*r*) was utilized in order to investigate the potentially significant relation between continuous variables such as COVID-19-related fear, depression, anxiety, and insomnia.

Finally, the Shapiro–Wilk test was used to evaluate whether the data were normally distributed and to test for data normality. Subsequently, a multivariable logistic regression model was used to investigate the factors, such as sociodemographic factors, mental health issues, and fear related to COVID-19 (independent variables), which may predict insomnia (a dependent variable).

## 3. Results

With a response rate of 96.6%, 145 students completed the study scales (145 out of 150). The average age of the participants was 21.6 years (SD: 0.63). The majority of participant 111 (76.6%) were between the ages of 18 and 22; 134 (92.4%) were unmarried; 36 (24.8%) did not receive support from their families; and 104 (71.7%) had a family monthly income of SAR ≥ 10,000. Clinically, 91% had no prior history of a chronic illness (Table 1). 

There were 79.3%, 30.2%, and 35.2% of students who reported fear related to COVID-19, depression, and anxiety, respectively. Approximately 24.7% of students claimed they had insomnia. Depression, anxiety, COVID-19-related fear, and insomnia were present in 30.6%, 38.7%, 80.2%, and 27.0% of the 18–22 age group, respectively.

Compared with single participants, married participants displayed increased symptoms of depression (45.5%), but lower levels of anxiety (36.4%), fear related to COVID-19 (72.7%), and insomnia (27.3%). Significantly lower levels of anxiety (18.3), insomnia (15.6%), and depression (26.6%) were reported by participants with family support compared with those without family support. Depression (46.2%), anxiety (38.5%), fear related to COVID-19 (92.3%), and insomnia (30.8%) were more prevalent among participants with chronic illness than among those without chronic illness.

The data in Table 2 show that higher levels of insomnia were associated with a significant increase in the age of the participants (*t* = −3.02, *p* = 0.003). Moreover, there was not a discernible difference seen for insomnia between married and single participants (*t* = −0.46, *p* = 0.0687). Those without family support were more likely to suffer from insomnia (*t* = −3.12, *p* = 0.003). Higher rates of insomnia were observed among students who suffered from chronic illnesses (*t* = −2.01, *p* = 0.002), but no significant differences in terms of family monthly income were identified (*t* = 0.27, *p* = 0.787).

The correlations between insomnia and depression, fear related to COVID-19, and anxiety are shown in Table 3. It was shown that anxiety (*r* = 0.302, *p* ≤ 0.001) and COVID-19-related fear (*r* = 0.284, *p* ≤ 0.001) were all significantly correlated with insomnia. However, a significant inverse association was observed between depression and insomnia (*r* = −0.216, *p* ≤ 0.001), considering that depression is assessed inversely with WHO-5, which means that a lower score indicates a larger degree of depression.

According to the binary logistic regression results, age, marital status, family income, and support from family did not significantly influence insomnia. Instead, depression, anxiety, fear related to COVID-19, and the presence of chronic illnesses did (Table 4). With rising anxiety, the probability of insomnia increased (AOR = 1.08, 95% CI: 0.92–0.97, *p* < 0.001), but decreased with increasing depression because WHO-5 measures depression in an inverted manner; a lower score suggests a greater degree of depression. As depression diminished, so did insomnia. (AOR = 0.87, 95% CI: 0.85–0.91, *p* < 0.001), and fear related to COVID-19 had been a predictor (AOR = 0.96, 95% CI: 1.07–1.21, *p* < 0.05). Chronic illness was also identified as a predictor of insomnia (AOR = 1.50, 95% CI = 1.01–2.24, *p* < 0.05). 

## 4. Discussion

This study estimated the prevalence of depression, fear related to COVID-19, anxiety, and insomnia among female nursing college students, as well as any possible predictors of insomnia. Recent studies that were carried out on college students all over the world during the COVID-19 pandemic indicated that there was a significant rise in the prevalence of mental health problems such as depression, fear associated with COVID-19, anxiety, and insomnia [58,59,60]. 

### 4.1. The Prevalence of Study Variables

Results from the present study confirmed prior research findings concerning the prevalence rates of anxiety, depression, fear related to COVID-19, and insomnia among female nursing college students, which were 35.2%, 30.2%, and 79.3%, respectively. This was comparable to the anxiety rate among nursing students in Japan, which was 34.6% [13], and significantly higher than the levels of anxiety among nursing students in Mongolia, which was 9.1%. Likewise, it was higher than in Saudi Arabia (7.3%) [61] and Italy (18.7%) [62]. In addition, the prevalence was lower than in Bangladesh (87.7%) [63] and Jordan (67.7%) [64]. The method used to evaluate anxiety, the study population, and the sociocultural differences between countries may all be potential explanations for the observed disparities.

The prevalence of depression in the current study, 30.2%, is comparable to findings from another study carried out in Malaysia, which indicated that 33.6% of the students had depression [13]. There were 36.4% of students studying health sciences who showed depressive symptoms, according to a study conducted in a comparable environment [65]. Although the prevalence in the present study was slightly lower than it was in a Malaysian study [65], it should be emphasized that our sample solely included students studying nursing. A different study found that 285 nursing students in Italy during the COVID-19 pandemic had a greater prevalence of depression (45.3%) than the current results [66]. 

In the present study, the prevalence of fear related to COVID-19 was 79.3%. In a similar vein, a study found that that students majoring in health sciences (such as nursing, medicine, etc.) scored more highly on the COVID-19 fear scale than other student groupings, and it was discovered that female nursing students exhibited higher levels of COVID-19-related fear [67].

According to the findings of the present research, 24.7% of the female nursing college students reported having problems sleeping. This result is consistent with those found in earlier studies, in which nursing students in colleges reported a prevalence of 26.6% for insomnia [68]. A prior meta-analysis estimated a weighted mean prevalence of insomnia among university students to be 18.5% (95% confidence interval [CI], 11.2–28.8%) [69].

### 4.2. Predictors of Insomnia

The results of the logistic regression demonstrate that depression, anxiety, fear related to COVID-19, and the existence of chronic illness had a substantial impact on students’ insomnia. On the other hand, when depression levels decreased, insomnia symptoms improved. Similarly, insomnia was significantly related to depression [70]. Additionally, among college students, insomnia has been linked to a variety of mental health problems in a number of research studies such as depression and anxiety [23,70,71].

However, when students’ fear of the COVID-19 and anxiety decreased, their insomnia reduced. Prior studies have explained the comparable results, stating that the fear of contracting the virus is responsible for 44% of the signs and symptoms of insomnia [72]. This is because anxiety and continual fear generate a significant disruption in cognitive function, which, in turn, impacts the sleep. The current outcome also matches that of an adult population [73,74]. Furthermore, one study found that the prevalence of insomnia and its associated components revealed a strong correlation between insomnia and fear related to COVID-19 (*p* < 0.001) [75].

The existence of chronic illness was found to be a significant factor in predicting a high level of insomnia. This findings are comparable to those of another study that also found a significant connection between chronic illness and insomnia [76].

In contrast to earlier research, which found that the risk of experiencing insomnia was 2.31 times greater among participants who had little family support compared with those who had good family support [77], family support was not a predictor of insomnia in the present study. It is possible that the rationale for this outcome is because the researcher in the current study did not use a reliable tool to measure family support. 

### 4.3. Limitations

Even though this study was intended to contribute to the body of knowledge and offer baseline data, when analyzing the outcomes of the study, it is absolutely necessary to consider the limitations. First, this study was cross-sectional, and participants were not followed up over time. If a long-term study was conducted, this limitation might be avoided; however, such a study would be both costly and time-consuming. Second, the inclusion of solely females in the sample reduced the generalizability of the results. Moreover, the participants in this research were undergraduates; thus, it is not possible to generalize the findings to cover all students in higher education. Third, because the data were based on self-rating measurements, recall bias may have been introduced. Thus, structured interviews are recommended. Fourth, no scale was used to measure the support provided by family members. Finally, the data collection for this study was conducted online, and consequently, only those participants who had access to the Internet and who already had some level of interest in the subject matter that was being studied were able to fill out the questionnaires. The degree of bias that may be present in online questionnaires cannot be determined with certainty [78]. As a result, it is recommended that the study be repeated, but this time the questionnaires should be administered on paper.

### 4.4. Implications

The above results show that mental health care providers need to learn more about the mental health problems that nursing students experience. The findings of this study are extremely important because they demonstrate that nursing students have a significantly increased risk of developing mental illnesses such as anxiety and depression. When these findings are considered, it is abundantly evident that the mental health of nursing students should be given the same level of attention as their physical well-being. Then, the students will be able to reflect on their issues and develop with constructive solutions when given this opportunity.

According to the findings of this study, there is a significant association between mental health issues, fear related to COVID-19, and insomnia. Due to this association, all of these problems need to be handled at the same time. It is highly recommended that nursing students seek quick support in order to feel less anxious, depressed, and afraid about COVID-19. Those nursing students who have significant levels of fear related to COVID-19, mental health problems, and insomnia may seek counseling in order to manage their mental health problems. The treatment may help them overcome their fears about the virus. 

Therefore, nursing students may receive psychological counseling for their mental health problems and support from psychiatric nurses. These nurses can help and support students’ psychological well-being by providing potentially appropriate strategies to help them cope with challenges under conditions that induce mental health problems. Additionally, nurse educators and academic advisers who actively engage with students have a responsibility to be aware of the potential risks during the COVID-19 pandemic. Furthermore, nursing students must be taught coping mechanisms, such as meditation and practices for stress management, in order to lessen the sense of anxiety and fear. The author suggests that decision-makers should take action to improve college students’ access to mental health services.

## 5. Conclusions

Depression, anxiety, insomnia, and fear related to COVID-19 are prevalent among female nursing college students. Significant predictors of the student’s insomnia level are depressive and anxious states, fears associated with COVID-19, as well as the presence of chronic illnesses. Due to the detrimental consequences that insomnia has on a variety of critical characteristics that are required for success in college, it is an issue that has to be addressed as soon as possible among college students. Problems with mental health have a crucial role in contributing to the widespread problem of insomnia. These findings shed light on how vital it is to include these factors in an intervention program that aims to minimize the occurrence of this health concern. In addition, it is necessary to conduct further research in order to track the development of this group and to determine measures that can be taken to reduce the prevalence of insomnia.

## Figures and Tables

**Table 1:** Description of socio-demographic characteristics and prevalence of study variables in relation to socio-demographic characteristics.

Variables	Total	(%)	Anxiety (A)		Depression (D)		Fear Related to COVID-19 (F)		Insomnia (I)	
	N	%	n	%	N	%	n	%	N	%
Overall	145	100	51	35.2	44	30.2	115	79.3	36	24.7
Age group (years)										
18–22	111	76.6	43	38.7	34	30.6	89	80.2	30	27
≥23	34	23.4	8	23.5	10	29.4	26	76.5	6	17.6
Family support										
Yes	109	75.2	21	18.3	29	26.6	91	83.5	17	15.6
No	36	24.8	30	83.3	15	41.7	24	66.7	19	52.8
Marital status										
Married	11	7.6	4	36.4	5	45.5	8	72.7	3	27.3
Single	134	92.4	47	35.1	39	29.1	107	79.9	33	24.6
Existence of chronic illness										
Yes	13	9	5	38.5	6	46.2	12	92.3	5	30.8
No	132	91	46	34.8	38	28.8	103	78	31	23.5
Family Monthly Income										
SAR < 10,000	41	28.3	17	41.5	20	48.8	32	78	13	31.7
SAR ≥ 10,000	104	71.7	34	32.7	24	23.1	83	79.8	23	22.1

**Table 2:** Descriptive and bivariate statistics of insomnia, with respect to participants’ characteristics (n = 145).

Variable	Insomnia		
	Mean (SD)	*t*	*p*
Age group		−3.02	0.003
18–22	8.12 (5.18)		
≥23	9.01 (6.51)		
Marital status		0.46	0.687
Married	8.45 (5.61)		
Single	8.43 (6.92)		
Family support		−3.12	0.003
Yes	8.32 (5.17)		
No	8.91 (6.85)		
Family Monthly Income		0.27	0.787
SAR < 10,000	9.25 (5.17)		
SAR ≥ 10,000	9.10 (6.85)		
Existence of chronic illness		−2.01	0.002
Yes	8.23 (5.19)		
No	8.91 (6.52)		

**Table 3:** Correlations between insomnia and study variables.

Variables	*R*	*p*
Anxiety	0.302	<0.001
Depression	−0.216	<0.001
Fear related to COVID-19	0.284	<0.001

**Table 4:** Factors associated with insomnia among female nursing college students using binary logistic regression (n = 145).

Variables	VIF	SE	*B*	AOR	*p*	95% CI	
						Lower	Upper
Depression	1.127	0.010	−0.119	0.87	<0.001	0.85	0.91
Fear related to COVID-19	1.426	0.014	0.025	0.96	0.020	1.07	1.21
Anxiety	1.10	0.013	0.079	1.08	<0.001	0.92	0.97
Age group (years)	1.035	0.135	0.076	1.07	0.571	0.82	1.42
Marital status	1.006	0.138	−0.035	0.95	0.793	0.73	1.27
Family support	1.033	0.127	0.067	0.85	0.591	0.86	0.90
Family monthly income	1.007	0.139	−0.034	0.92	0.749	0.74	1.26
Existence of chronic illnesses	1.021	0.201	0.414	1.50	0.030	1.01	2.24

## Data Availability

The author will, upon receiving a reasonable request, make the datasets that were used in this study available.
